# Current advances and future trends of hormesis in disease

**DOI:** 10.1038/s41514-024-00155-3

**Published:** 2024-05-15

**Authors:** Yantong Wan, Jinxi Liu, Yiyin Mai, Yinghao Hong, Zixuan Jia, Guijie Tian, Yunzhuo Liu, Huaping Liang, Jinghua Liu

**Affiliations:** 1 State Key Laboratory of Trauma and Chemical Poisoning, Army Medical University, Chongqing, China; 2 Guangdong Provincial Key Laboratory of Proteomics, Department of Pathophysiology, School of Basic Medical Sciences, Southern Medical University, Guangzhou, Guangdong China; 3 School of Laboratory Medicine and Biotechnology, Southern Medical University, Guangzhou, Guangdong China; 4 The Second School of Clinical Medicine, Southern Medical University, Guangzhou, Guangdong China

**Keywords:** Risk factors, Stress and resilience

## Abstract

Hormesis, an adaptive response, occurs when exposure to low doses of a stressor potentially induces a stimulatory effect, while higher doses may inhibit it. This phenomenon is widely observed across various organisms and stressors, significantly advancing our understanding and inspiring further exploration of the beneficial effects of toxins at doses both below and beyond traditional thresholds. This has profound implications for promoting biological regulation at the cellular level and enhancing adaptability throughout the biosphere. Therefore, conducting bibliometric analysis in this field is crucial for accurately analyzing and summarizing its current research status. The results of the bibliometric analysis reveal a steady increase in the number of publications in this field over the years. The United States emerges as the leading country in both publication and citation numbers, with the journal Dose–Response publishing the highest number of papers in this area. Calabrese E.J. is a prominent person with significant contributions and influence among authors. Through keyword co-occurrence and trend analysis, current hotspots in this field are identified, primarily focusing on the relationship between hormesis, oxidative stress, and aging. Analysis of highly cited references predicts that future research trends may center around the relationship between hormesis and stress at different doses, as well as exploring the mechanisms and applications of hormesis. In conclusion, this review aims to visually represent hormesis-related research through bibliometric methods, uncovering emerging patterns and areas of focus within the field. It provides a summary of the current research status and forecasts trends in hormesis-related research.

## Introduction

In the early 16th century, Swiss-German physician Paracelsus (1493–1541) introduced the following concept: “All things are poison, and nothing is without poison, only the dose permits something not to be poisonous” (“Sola dosis facit venenum”)^1^. Later, it was gradually discovered that biphasic dose–response appeared widely in various fields. This response, characterized by stimulation at low doses and inhibition at high doses, was later called the concept of “hormesis”^2,3^. Researchers now recognize that the essence of^4^ hormesis is an adaptive response^5^. The response outcome exhibits a non-linear relationship with the different doses of stimulation, and its dose–response model is biphasic^6^. Indeed, under the push of a stressor at low doses, the stimulus source can positively promote or regulate the physiological functions of organisms by inducing the reconstruction of homeostasis. However, at high doses, the disruption of balance and the lack of compensation in the organism can lead to harmful effects^7^.

Hormesis has universal applicability and a high degree of generalizability^8^. The former refers to the discovery of hormesis in various physical stressors such as temperature^1,9^, ionizing radiation^10^^,^ energy deficiency^11^, low nitrogen^12^, or chemical stress^13^ like ROS (reactive oxygen species)^14^, herbicide^12,15^, curcumin^16^, antibiotics^17^, trace element or heavy metal^18–24^, and also biological stressors. And hormesis can also elicit biological effects at multiple levels, including cellular, organ, individual, and population^25^. These responses were observed in various microbial, plant, and animal models, indicating their extensive applicability^26–28^. In this context, generalizability suggests that the existence of hormesis may be more widespread than currently defined in databases such as Web of Science or PubMed. Researchers like Calabrese E.J. and Blain have established the hormesis database, which includes over 9000 hormetic models^29^. We anticipate that in the future, more concepts will be recognized and incorporated into the scope of hormesis.

The hormesis mechanism is highly complex and cannot be explained by a single biological theory. However, researchers have been able to summarize some general patterns preliminarily. Hormesis can be activated by signaling pathways such as NF-κB (nuclear factor kappa-B)^30^, MAPK (mitogen-activated protein kinase)^31^, AMPK (Adenosine 5‘-monophosphate (AMP)-activated protein kinase), mTOR (mammalian target of rapamycin), PI3K/Akt (Phosphatidylinositide 3-kinases/protein kinase B)^32^, an upregulation of cytoprotective proteins, including phase 2 enzymes, antioxidant enzymes, heat-shock proteins, growth factors, and proteins involved in the regulation of cellular energy metabolism^14,33,34^. Consequently, this helps in diminishing oxidative stress and inflammation levels, mitigating cellular senescence, and influencing the emergence of the aging phenotype^32^. This process allows for tissue repair, restoration of cell homeostasis, and improvement of the body’s defense and adaptability. As a result, it can prevent or reduce damage caused by subsequent higher doses of stimuli and enhance overall health and biological performance throughout the lifespan^16^.

**Fig. 1:**
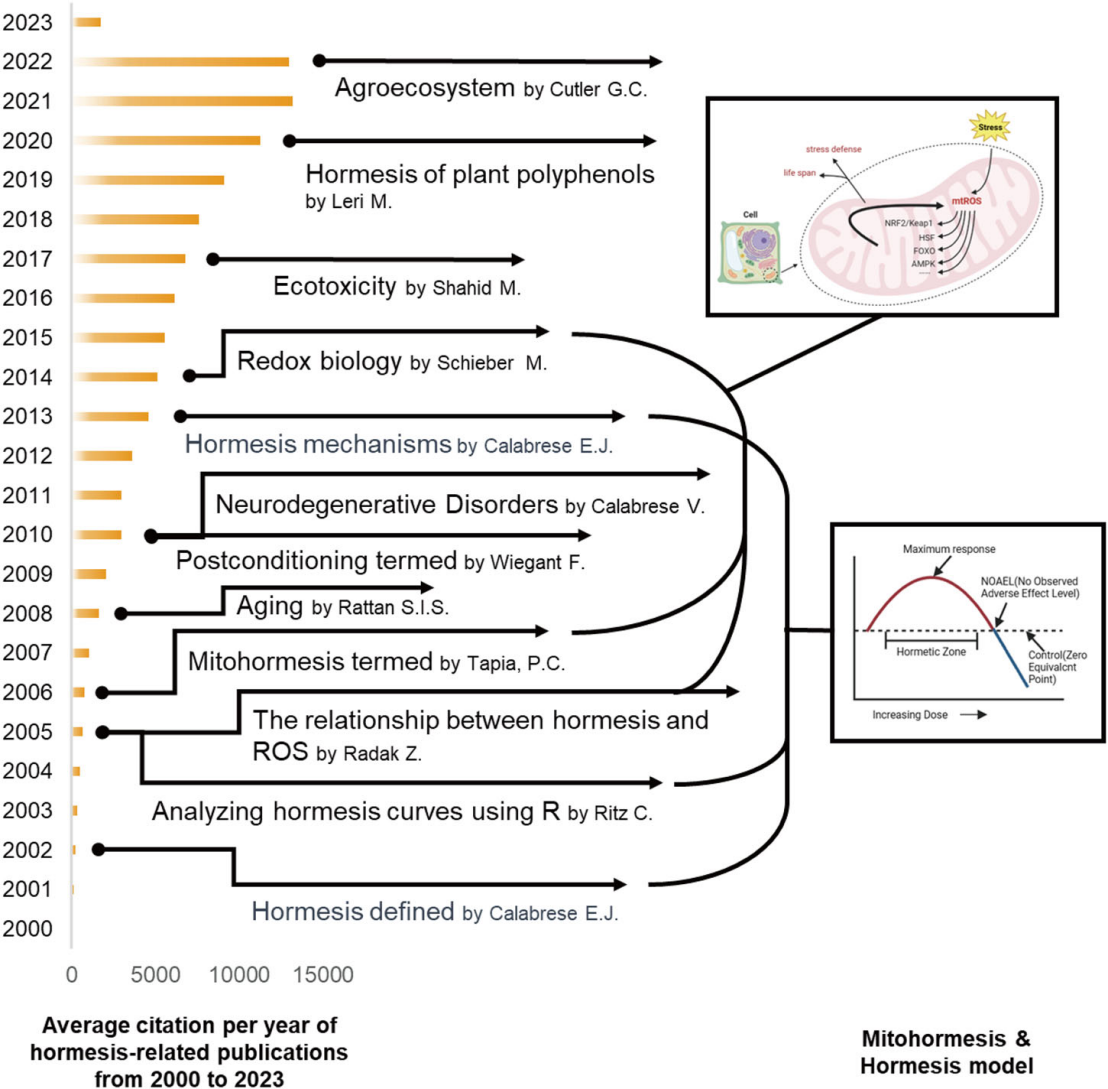
The developmental history of hormesis and the temporal milestones of significant scientific discoveries.

Therefore, researchers can grasp and assess the quantitative characteristics of hormesis, including the stimulation’s magnitude, the stimulus’s width, and their relationship to the zero equivalent point^35^. Hormesis, by defining the limits of biological plasticity, holds significant implications for various areas such as drug development, disease resistance, and strategies to enhance biological performance and resilience. This underscores the importance of utilizing techniques such as pre-and post-conditioning in the fields of neuroscience^36^, immunology^37^, tumor cell biology^38^, wound healing^39^, biogerontology^40^, plant biology^41^, or other major biomedical content areas to achieve greater resilience^42,43^. These findings have significant implications for designing studies and exploring commercial products in clinical, laboratory, and field settings^44^ (Fig. 1).

The controversial status and value of hormesis in the past have transformed, and the actual value of this ancient field is gradually being recognized. However, there still needs to be a more comprehensive analysis and macroscopic description of the hormesis field^45^. This article aims to utilize CiteSpace and VOSviewer for a more in-depth visual analysis of hormesis-related articles. This approach provides researchers with an expanded comprehension of the field and facilitates the exploration of future research directions.

## Results

### Annual publications and citation trends

**Fig. 2:**
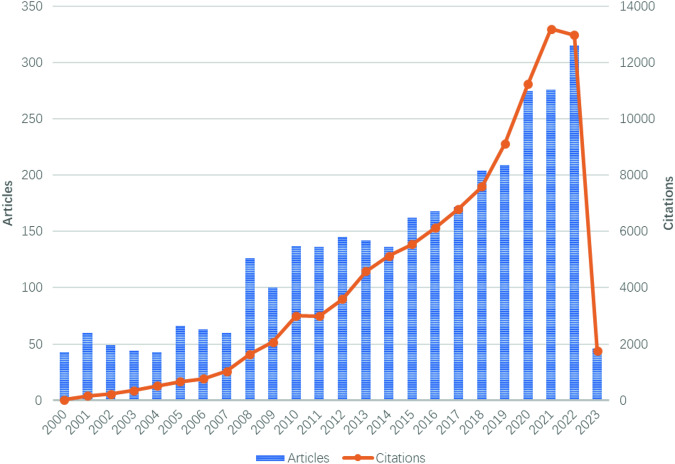
The combination chart presents the annual publication count and average citation per year of hormesis-related publications from 2000 to 2023. Both the annual publication citation frequency shows an overall increasing trend year by year. The number of publications shows a general upward trend with a peak in 2022. In the last decade (2012–2021), the average annual publication count exceeds 240. Due to the data collection for the year 2023 being recorded until March 31, 2023, the included data for this year is relatively limited compared to other years.

The bar chart in Fig. 2 illustrates the yearly publication volume of articles related to the subject from 2000 to 2023. The overall trend in the publication volume of this field has been increasing since the beginning of the 21st century, with bursts in the number of publications occurring in 2008 and 2020. The year with the highest number of publications is 2022 (2023 data only until March 31), with 315 articles.

The line graph shows related articles’ annual average citation frequency from 2000 to 2023. Overall, the annual citation frequency demonstrates a consistent upward trend, even when the publication volume for a given year did not experience growth or decline. Notably, there was a significant increase from 2018 to 2021, reaching its peak of 13,196 citations in 2021.

### Distributions of countries/regions and institutions

Supplementary Dataset 1 presents the top 10 countries/regions in terms of publication count, along with their citation frequency and total link strength. The United States is the dominant country in terms of the number of publications, publishing the most documents (894), followed by China (653), Italy (236), Germany (201), and all other countries with less than 200 publications. Notably, the USA accumulated a total of 43,321 citations, which is nearly four times higher than China’s 11,645 citations. The United States consistently ranks in all three categories, demonstrating its significant influence in this field. The United States leads regarding the number of articles published and their quality. Similarly, in the analysis of institutions (Supplementary Dataset 2), the University of Massachusetts, USA, stands out with the highest number of publications (226) and the highest citation count (14,718), surpassing other institutions. This suggests that the University of Massachusetts has had a significant impact in terms of the quantity and quality of articles published in the field of hormesis.

**Fig. 3:**
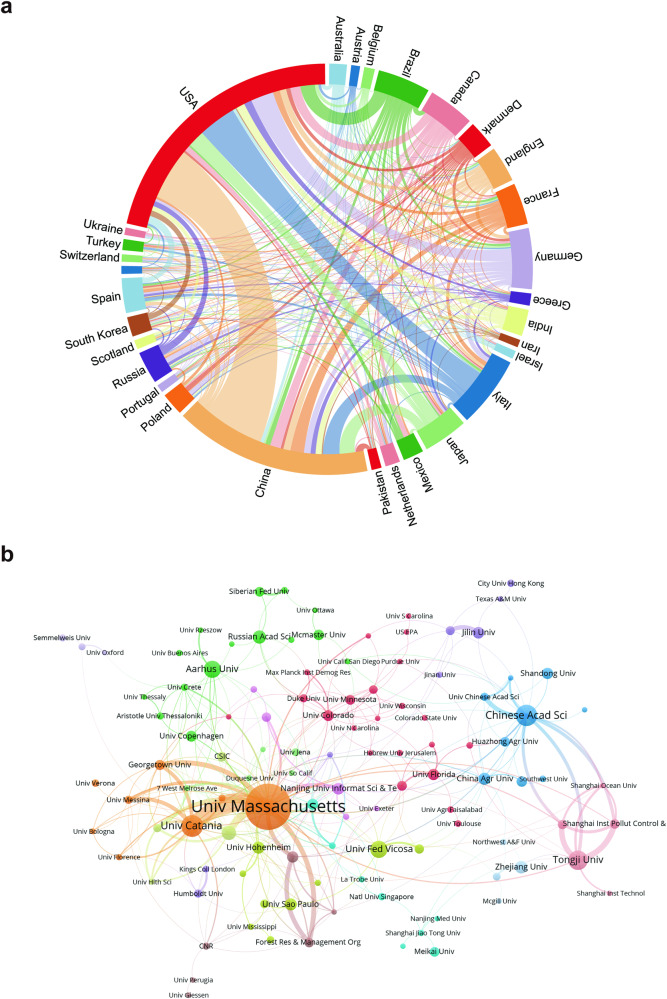
The collaborative network of countries/regions and institutions. **a** The chord diagram illustrates the cooperative relationships between countries in the field of hormesis research. The lines’ width represents the connections’ strength, with wider bands indicating stronger collaborations. Countries with wider bands and more diverse colors on the lines indicate that they have established collaborations with more countries. **b** The collaborative network of institutions was visualized using VOSviewer. The University of Massachusetts occupies a central position in the collaborative map of institutions, indicating its significant influence. This map visualizes the cooperative relationships and correlations of research between institutions.

Figure 3a illustrates the strong collaboration between various countries in the field of hormesis. The closest collaboration is observed between the United States and China, followed by the United States and Italy. These countries with closer collaborative relationships are also influential in the hormesis field. The connections among these countries also contribute to the robust development of hormesis research and have a mutually beneficial effect. Figure 3b, generated using VOSviewer, explores the global distribution of hormesis research and identifies potential collaboration opportunities among research institutions. The collaboration among institutions was categorized into eight closely associated clusters or groups, reflecting the level of collaboration and interconnectedness in the field of hormesis research. The University of Massachusetts and the University of Catania, leading publication and citation volume institutions, exhibit strong connections, indicating a robust collaborative relationship. Secondly, there are links with Georgetown University and The University of British Columbia (Forest Resources Management Department). Among Chinese institutions, institutions with strong citation relationships are mainly between the Chinese Academy of Sciences and Tongji University. In addition, some institutions only have relatively single links with the outside world. These institutions are relatively independent of other institutions, such as Semmelweis University and the University of Oxford, the University of Perugia and the University of Giessen, the City University of Hong Kong, and Texas A&M University.

### Distribution of authors and co-cited authors

Supplementary Dataset 3 displays the top 10 authors ranked by the number of publications, co-citation frequency, affiliated institutions, and total link strength^46^. The author with the most publications is identified as Calabrese, Edward J. (224) from the University of Massachusetts, followed by Agathokleous, Evgenios (74) from Nanjing University of Information Science and Technology, and Calabrese, Vittorio (56) from the University of Catania. It is worth noting that the most frequently co-cited and cited author in this field is Calabrese, Edward J., who powerfully demonstrates his significant influence in the field and contributed to the highest citations and publications of the University of Massachusetts.

**Fig. 4:**
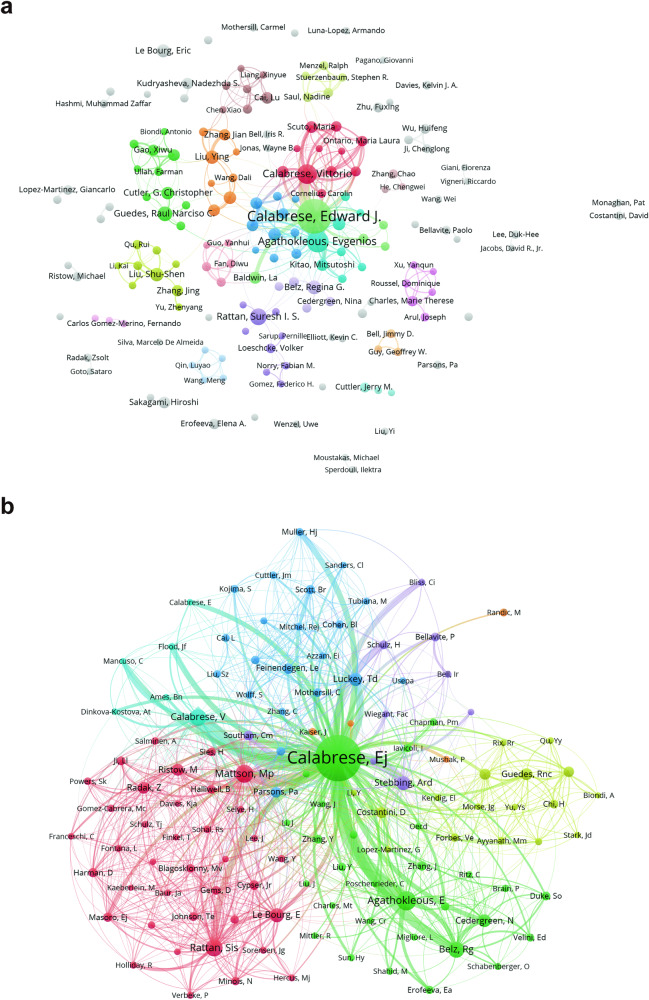
The collaborative network of co-authors and co-cited authors was visualized using VOSviewer. **a** The co-authors’ map of hormesis research provides a visual representation of the relationships between authors involved in the study. Each node, represented by a different color, corresponds to an author belonging to a specific cluster. The size of each node indicates the frequency of co-occurrence, while the connections between nodes represent the co-occurrence relationships among authors. **b** In the co-cited authors’ map of hormesis research, E.J. Calabrese occupies a central position, indicating their significant influence in the field. This map visualizes the co-cited relationships between authors, highlighting the similarities in their research interests and contributions.

In Fig. 4a, a cooperation network is depicted among authors, serving as a valuable resource for identifying potential research collaborators. Like the above analysis of countries/regions and institutions, the network is visually represented using 18 distinct colors, each representing a different cluster of authors within Fig. 4a. Edward J. Calabrese plays a central role in the collaborative network, serving as the most critical contributor to the hormesis study. Collaboration among authors, particularly those within the same cluster, such as Rual Narciso C. Guedes and G. Christopher Cutler, is evident as they work together on the research. Authors from different clusters, including Edward J. Calabrese with Vittorio Calabrese and Evgenios Agathokleous, have also demonstrated close collaboration, indicating active cooperation between different research domains such as toxicology, biology, and environmental studies.

Co-cited authorship analysis involves investigating situations where a third author references two authors. A greater frequency of co-citations suggests a more vital similar academic interest and research focus^47^. By examining the authors with the highest number of publications and co-citation frequencies in hormesis-related research, we can gain valuable insights into their research expertise and identify the significant areas of study within the field of hormesis research. The authors depicted in Fig. 4b were predominantly grouped into 7 clusters. On the whole, the research content within this field exhibits a high degree of correlation, especially in the orange cluster where M. Randic is situated, which also demonstrates connections with several other clusters. The remaining six clusters are E. J. Calabrese, E. Agathokleous, etc. (green) in the central position; M. Ristow, Mp. Mattson, etc.(red); V. Calabrese, etc. (light blue); Td. Luckey, etc. (dark blue); H. Schulz, Ard. Stebbing, etc.(purple); Rnc. Guedes, etc. (yellow).

### Distribution by journals

**Fig. 5:**
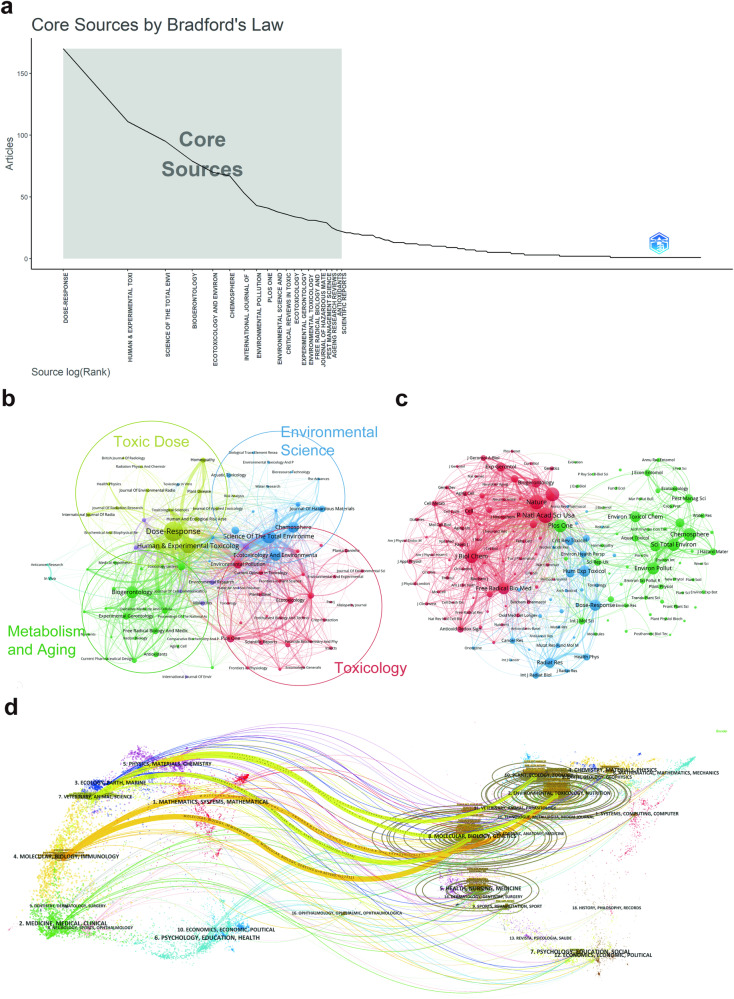
The analysis of academic journals related to hormesis. **a** Bradford’s Law can be applied to academic journals in the field, as evidenced by the gray-shaded area in the figure. This area encompasses the journals that have made significant contributions to the field, arranged in descending order based on the number of articles. The pattern observed in this gray-shaded area aligns with the findings presented in Supplementary Dataset 5. **b** The visualization of journal collaborations in VOSviewer analyzes the collaborative relationships between journals. In the figure, journals that have published more than ten documents are depicted as nodes of varying colors, representing their membership in different clusters. The size of the nodes corresponds to the frequency of these journals’ presence in the network. **c** The visualization of journal citations in VOSviewer allows for exploring the connections between journals. The size of the nodes signifies the frequency of their citations, indicating the importance and influence of the journals within the network. **d** The dual-map overlay of journals illustrates the citation relationships between citing journals on the left and cited journals on the right. The width of the connecting lines signifies the strength of the citation relationships.

The visual analysis examined the active and influential journals in hormesis research based on published articles and co-citation patterns. According to Supplementary Dataset 4 and Fig. 5a, the journal with the highest number of publications is Dose–Response (170), followed by Human and Experimental Toxicology (111), Science of the Total Environment (95), and several other journals with fewer than 80 publications. Among the top 10 journals in hormesis research, six are categorized as Q1 in the Journal Citation Reports (JCR2022), indicating their high impact and influence in the field. Additionally, six of these journals have an impact factor (IF) of over 5^48^, further highlighting their significance. The citation counts of the top ten co-cited academic journals were distributed around an average of 2000, indicating a substantial level of citations and recognition in the field. However, it is worth noting that while prolific journals frequently represent this area’s research history and status quo, highly cited journals could herald its broader and more promising paths forward. Over half of the ten most co-cited journals rank in the first quartile, and the top three—PNAS, Nature, and Science—are extremely prominent interdisciplinary journals. This hints that hormesis’ promise across domains has attracted researchers’ eyes and excitement.

The visualization generated using VOSviewer illustrates the journals where hormesis-related literature has been published and the interrelationships between these journals. The clustering of journals is based on their similarities, resulting in five distinct categories (Fig. 5b). The red cluster primarily consists of journals with a focus on toxicology (Environmental Pollution, Ecotoxicology, Plos One, etc.); the green cluster is mainly focused on metabolism and aging (Biogerontology, Oxidative Medicine and Cellular Longevity, etc.); the yellow cluster is mainly about a toxic dose (Human and Experimental Toxicology, Dose–Response, etc.); the blue cluster is focused on environmental science (Science of The Total Environment, Ecotoxicology and Environmental Safety, etc.); the purple cluster is focused on cell biology (Molecules, Journal of Cell Communication and Signaling).

These journals have been categorized into three clusters based on their co-citation frequency in Fig. 5c, indicating that they tend to focus on similar research directions. The red cluster is mainly focused on cells, biology, and medicine (Nature, Proceedings of the National Academy of Sciences of the United States of America, Free Radical Biology and Medicine, etc.); the green cluster is mainly in the field of environmental science (Chemosphere, Science of the Total Environment, Environmental Pollution, etc.); the blue cluster focused on toxicology and disease (Human and Experimental Toxicology, Dose–Response, etc.).

Figure 5d presents a dual-map overlay of journals, providing a visual representation of the distribution of academic journals, the evolution of citation patterns, and the changes in research emphasis among these journals. This overlay allows for a comprehensive understanding of the relationships and dynamics within the field of hormesis research, highlighting the evolving landscape of scholarly publications and their citation patterns. On the dual map, journals that cite other journals are shown on the left, while the journals that are being cited are shown on the right. A colored line connects a citing journal on the left to a cited journal on the right, indicating the direction and context of the citation. The citing journals predominantly belong to molecular biology, immunology, veterinary science, animal science, and other research frontiers. On the other hand, the cited journals mainly come from molecular biology, genetics, environmental science, toxicology, nutrition, plant science, ecology, and zoology.

### Keywords analysis

Keywords are crucial in representing research articles’ main themes and content. Analyzing the co-occurrence of keywords provides valuable insights into the focus and trends of research within a specific field. By identifying frequently co-occurring keywords, we can uncover prominent themes, conceptual relationships, and emerging areas of interest.

Supplementary Dataset 5 displays the top 20 keywords in hormesis research based on their frequency of occurrence. The keyword “hormesis” appears most frequently (1485), followed by “oxidative stress” (200), aging (185), and dose–response (150), which may indicate the mechanisms, characteristics, and widespread application directions of hormesis.

**Fig. 6:**
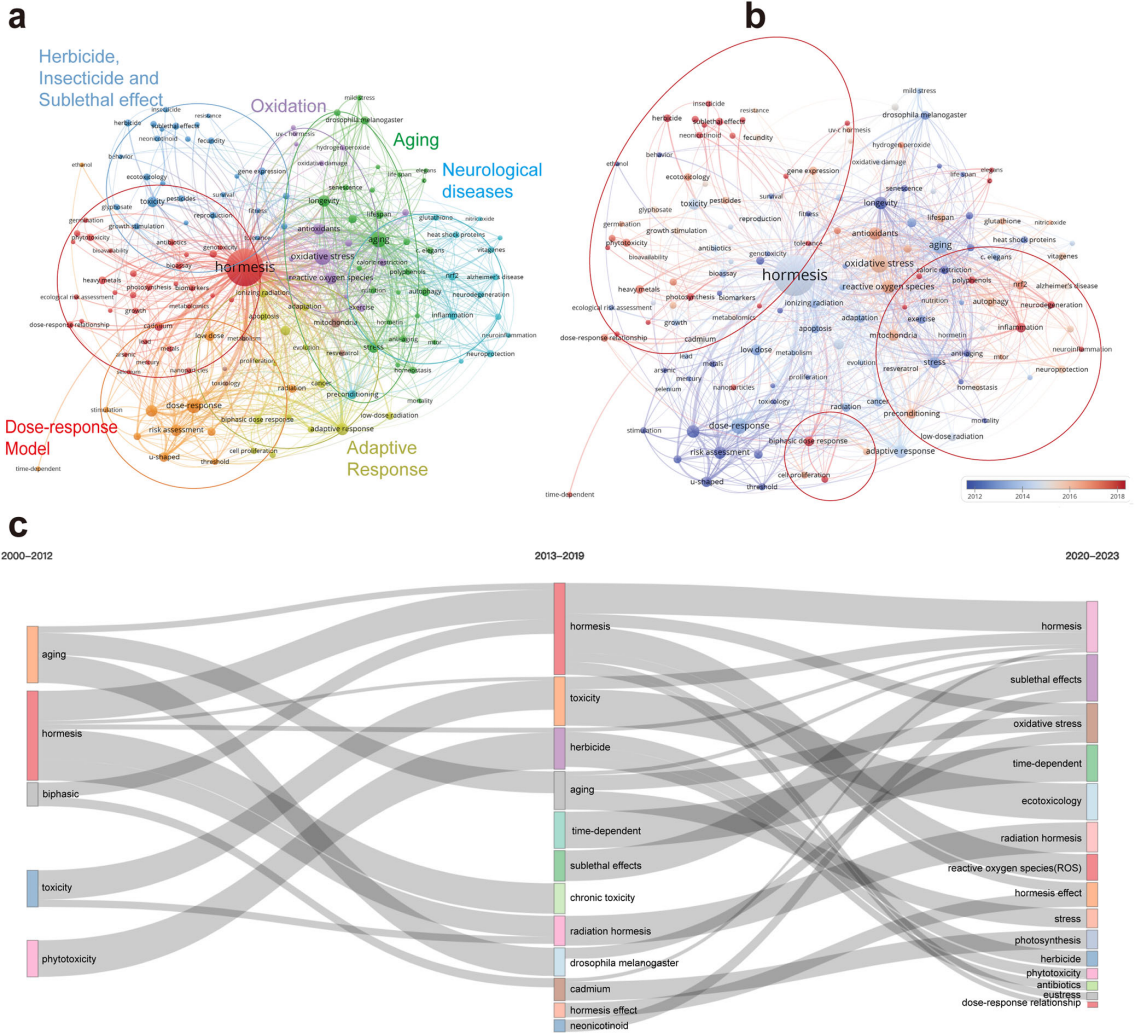
The analysis of keywords related to hormesis. **a** The co-occurrence keywords’ map of hormesis research visualizes the relationships between keywords involved in the study. The nodes, represented by different colors, correspond to keywords of different clusters. The size of each node indicates the frequency of co-occurrence, while the connections between nodes represent the co-occurrence relationships among keywords. **b** The figure displays the ratio of keywords to the total number of keywords in the past five years, reflecting the relative contribution of each institution to hormesis research compared to their overall contribution from 2000 to 2023. The color scale in the figure represents this ratio, with a red bias indicating a higher ratio. This indicates that institutions with a red bias are emerging as influential contributors in the field of hormesis. Conversely, a blue bias suggests a lower ratio, indicating that these institutions have conducted less research on hormesis in recent years. **c** The chronological Sankey diagram depicts the developmental trends and interconnections of keyword popularity over time. The width of the bands and connecting lines is proportional to their respective popularity and correlation strength. It is important to note that the years covered by the three time periods are not uniform but decrease in sequence according to their temporal distance. This is done to present more accurate and valuable recent trends.

Using VOSviewer, the keywords were analyzed and classified into eight closely related clusters in Fig. 6a, indicating the level of collaboration and interconnectedness among different keywords in the field of hormesis research. Figure 6b presents the ratio of keywords to the total number of keywords in the past five years. We can deduce the association and research intensity between different keywords by analyzing the two figures. In the clusters (red, orange, and yellow) on the dose–response model of hormesis, emerging keywords are biphasic dose–response. Research hotspots in the application of hormesis include keywords related to inflammation and neurotic (light blue), herbicide and insecticide, and sublethal effect (dark blue). In addition, although research on aging, longevity, lifespan (green), and adaptive response (yellow) is not new, it is still a research focus of hormesis. There are also some relatively small clusters, such as mitohormesis. Although fewer keywords exist in this cluster, the links with other keywords are very close, and the intensity is quite good. This shows that this direction may have more excellent research value to be tapped. The Sankey diagram in Fig. 6c depicts the evolution of keywords over time, reflecting their development trends. In the early 21st century, the focus on hormesis was centered around its toxicity, dose–response characteristics, and its application in aging studies. Subsequently, the emphasis on toxicity-related research declined, and the relationship between hormesis, oxidative stress, and ROS started to emerge. The direction of sublethal effects, a refined aspect of biphasic effects, gained prominence, replacing the toxic effects at high doses as a significant feature and research focus of hormesis.

**Fig. 7:**
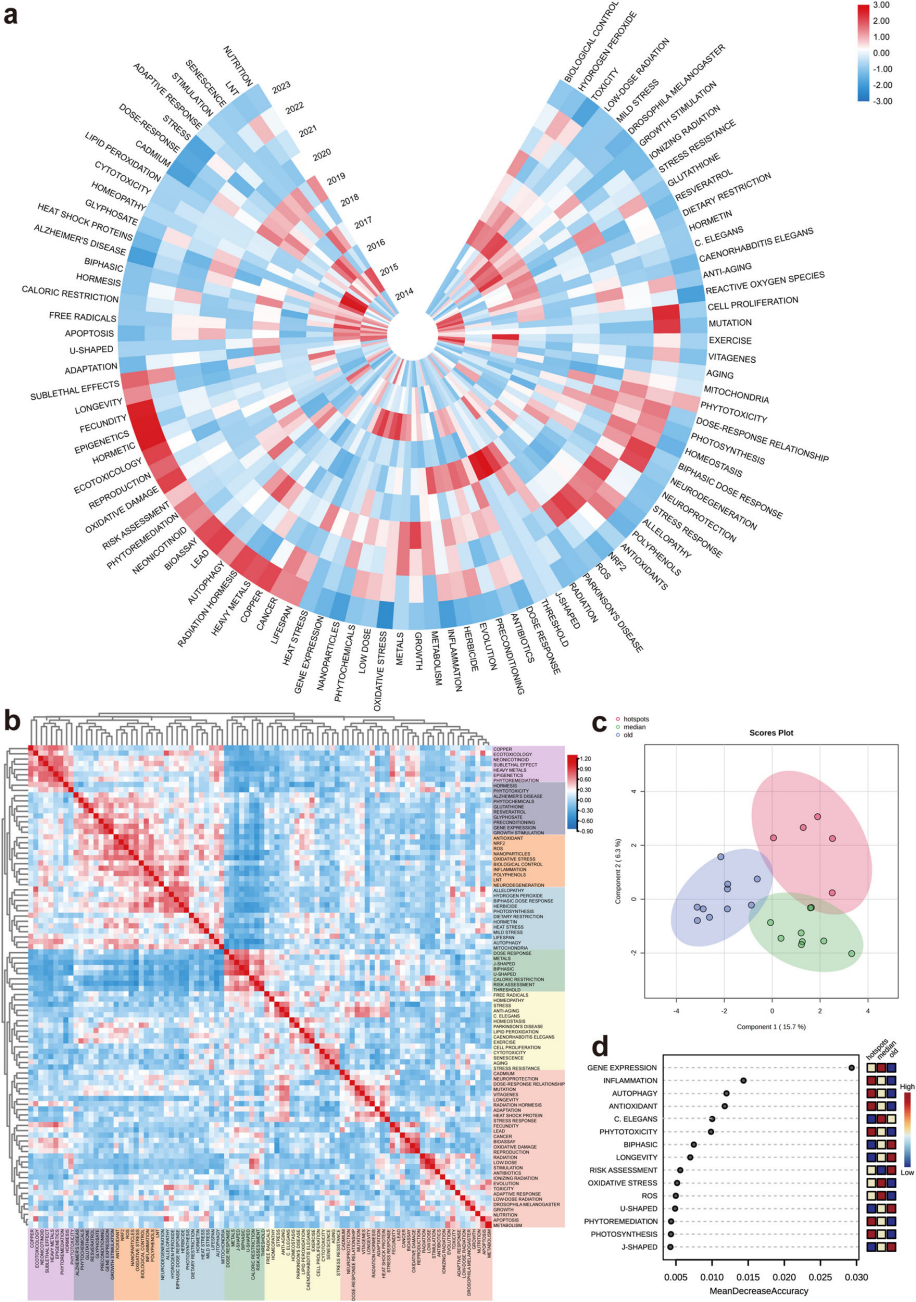
The analysis of keywords related to hormesis. **a** The annual heatmap of hormesis research illustrates the popularity of keywords between 2014 and 2023, measured by the ratio of yearly citation counts to total citation counts for each keyword. The red color indicates the highest levels of popularity. **b** The relevance heatmap of hormesis research illustrates the popularity of keywords. Keywords with high popularity during comparable periods are grouped into distinct categories and distinguished by varying colors. **c** The principal component analysis of hormesis research reveals the popularity of keywords. Keywords are categorized based on the year of occurrence and are represented by circles of three different colors. The distances between the individual points within each category indicate their relevance. **d** Conducting a heat analysis on keywords in the field of hormesis using the random forest method. The color distribution in the three columns to the right of each keyword row represents the dynamics of popularity over time.

The heatmap in Fig. 7a illustrates the trend of keyword popularity over time. Currently, highly popular keywords include epigenetics, mutation, and cell proliferation in the field of molecular biology. Keywords with significant attention in application areas include neurodegeneration, anti-aging, lifespan, and cancer, as well as concepts like dose–response relationship, sublethal effects, and hormetics. Conversely, keywords such as homeopathy, cytotoxicity, lipid peroxidation, and threshold have shown decreased levels of attention. Figure 7b displays the heat map of the correlation between keywords, with keywords of higher similarity marked in the same color, forming a total of seven colors. The purple cluster is mainly composed of toxicology-related concepts, such as ecotoxicology and sublethal effect; the gray cluster consists of botanical and medical terms, including hormesis, phytotoxicity, glyphosate, Alzheimer’s Disease, and preconditioning; the orange and blue clusters primarily include keywords in the field of molecular biology, such as NRF2 (Nuclear factor erythroid 2-related factor 2), ROS, oxidative stress, mitochondria, and heat stress; the green cluster lists dose levels; the green cluster lists dose levels in the field of molecular biology; the green cluster lists dose–response and its various models such as J-shaped, biphasic, and threshold; the yellow cluster accommodates many keywords in the field of cell biology, such as homeostasis, cytotoxicity, stress resistance, etc.; the red cluster in the bottom right corner gathers keywords from various fields, including medicine, physiology, genetics, molecular biology, such as cancer, adaptation, oxidative damage, evolution. In addition to further clarifying the temporal categorization of these keywords for trend analysis, they were manually classified into three categories: old (before 2010), median (2010–2018), and hotspots (2019–2023). The results of principal component analysis (PCA) are shown in Fig. 7c, indicating some overlap between the three groups but with apparent separation, demonstrating that the similarity within each group is not significant in the chronological development, proving the effectiveness of the classification, and indicating significant changes in the development and evolution of keywords in this field. Subsequently, the results of the analysis of keyword popularity using the random forest method, as shown in Fig. 7d, highlight the keywords with high popularity in the last five years, including inflammation, autophagy, antioxidant, phytotoxicity, phytoremediation, and photosynthesis.

### Highly cited reference analysis

Supplementary Dataset 6 shows the top 15 most cited articles about hormesis; the most frequently cited article is “ROS Function in Redox Signaling and Oxidative Stress” (Schieber, M; Chandel, NS^49^) (2357), wherein low doses of stimulation and high doses of inhibition are observed, and the term to describe this phenomenon is “hormesis.” This article illustrates the redox biology and oxidative stress caused by ROS and the potential to alleviate disease without interfering with healthy tissue^49^.

Among them, several highly influential authors in the field have contributed more than one highly cited article (Supplementary Dataset 6), such as Ristow, M. (three of his articles are among the top 15 most cited articles), Calabrese, E.J., and Gomez-Cabrera, MC (two of his articles are among the top 15 most cited article) have made a significant contribution in this field.

**Fig. 8:**
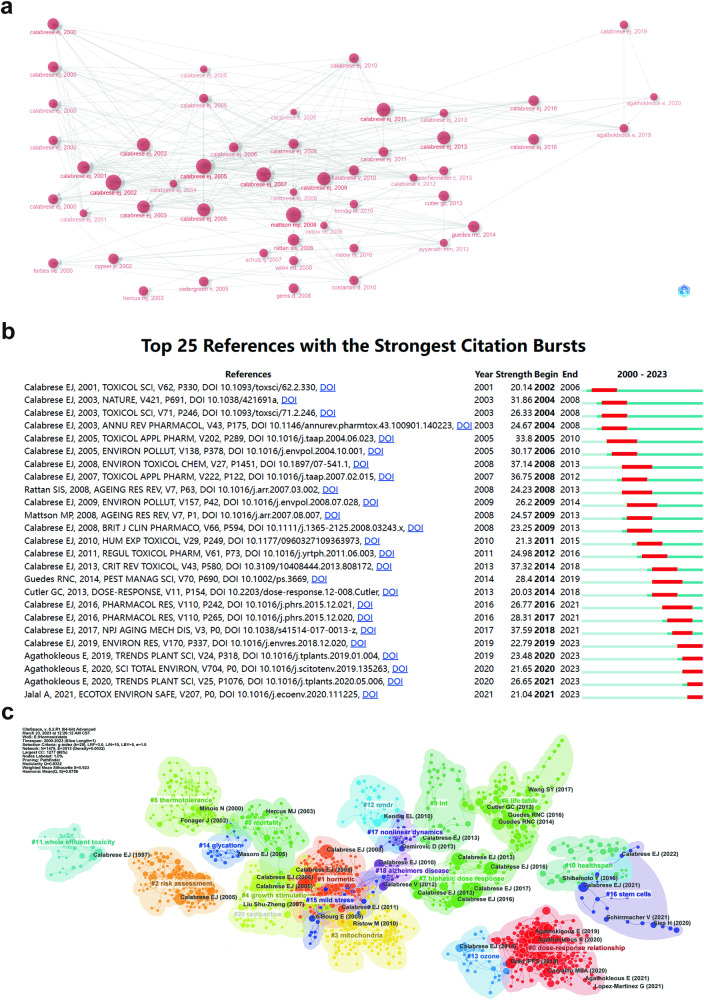
The analysis of highly cited references related to hormesis. **a** Association between the top 45 highly cited references. The size of each node indicates the intensity of the citation. The nodes are arranged from left to right in chronological order of citation occurrence, with the furthest right being the most recent cited. **b** The figure showcases the top 25 references with the most robust citation bursts, highlighting abrupt shifts in information represented by red outbreaks along the timeline. These bursts indicate rapid increases in citations and the emergence of essential questions or solutions in the field. The burst detection method by Kleinberg J in CiteSpace is used to identify these significant shifts. **c** Clustering of references based on the similarity between references, including #0 dose–response relationship, #2 risk assessment, #3 mitochondria, #4 growth stimulation, #5 thermotolerance, #6 life table, #7 biphasic dose–response, #8 mortality, #9 lnt, #10 healthspan, etc.

Figure 8a shows the association between these high-burst articles. The early cited articles were from Calabrese E.J. Between 2005 and 2010, Calabrese E.J. and Mattson M.P. mainly integrated these previous research progress and linked the next research stage. Finally, Agathokelous and Calabrese E.J. jointly summarized these conclusions in “A Global Environmental Health Perspective and Optimization of Stress”^50^. A significant and sudden increase in the number of citations over a specific period characterizes references with citation bursts. The first three citation bursts in the field of hormesis research began in 2003 (Fig. 8b). The most prominent burst, with a strength of 37.59, occurred in a paper titled “How does hormesis impact biology, toxicology, and medicine?” published in npj Aging and Mechanisms of Disease by Calabrese E.J., Edward J. in 2017, with citation burst from 2017 to 2021. Another article with a similar burst strength is “Hormesis: Why it is important to Toxicology and Toxicologists (strength=37.59)” in 2008 and “Hormetic Mechanisms (strength = 37.32)” in 2013, and these three papers share the same authors. According to the findings, 2008 had the highest citation bursts, followed by 2004. Notably, five references are still in the burst.

The relationships between studies were analyzed using CiteSpace, and Fig. 8c displays the authors and years of articles that exhibited a burst in citation frequency. and is categorized into 19 groups represented by different colors. The category labeled #0 has the highest number of published articles, and the most commonly occurring keyword in these articles is “dose–response relationship”. The category labeled #0 has the highest number of published articles, and the most commonly occurring keyword in these articles is “dose–response relationship”. In terms of the timeline, the earliest research area in the field of hormesis forms a distinct research cluster: #11 (whole effluent toxicity; S = 0.989;1997), and three other separate research clusters #5 (thermotolerance; S = 0.941; 1999), #8 (mortality; S = 0.972; 2003), and #14 (glycation; 0.955; 2004) are linked together. Meanwhile, cluster #2 (risk assessment; 0.92; 2001) is often discussed, and the most influential author in this cluster is also Calabrese E.J. Before 2005, research in this field had yet to delve deeply into its essence and its connection to diseases was limited. After it, with the rise of the concept of hormesis, there has been a growing recognition of the relationship between hormesis and stress or stimulation at different doses, as well as the mechanisms and application of hormesis, such as #3 mitochondria (0.869;2010), hormesis in mitochondria may be related to the beneficial effects of hormesis in delaying aging and treating diseases^51^. The most famous cluster, the #0 dose–response relationship (S=0.959; 2019), appeared around 2020 and elucidated the fundamental nature of hormesis as a dose–response relationship characterized by low-dose stimulation and high-dose inhibition in plants^15^.

## Discussion

In this bibliometric analysis, we utilized CiteSpace 6.1.R2 Advanced, VOSviewer 1.6.18, and R-bibliometrix to analyze 3177 research articles related to hormesis. These data were retrieved from the Web of Science Core Collection database between January 1, 2000, and March 31, 2023. The rise in annual published articles shows that this field has been gaining increasing prominence and interest over time. Calabrese E.J. et al. provided a systematic and comprehensive definition of the concept of hormesis in 2002, which has significantly contributed to enhancing our understanding of it. Research articles on hormesis have increased by more than tenfold since 2000. From 2000 to the present, publications related to hormesis have shown an overall increasing trend, indicating that the research interest in hormesis continues to grow. There were two significant spikes in publication volume within this field in 2008 and 2014. In 2007, the regulatory effect of glucose restriction on oxidative stress levels in Caenorhabditis elegans was identified^52^. A year later, the recognition of hormesis induced by exercise^53^ or alterations in cellular nutrition conditions in humans^54^ furthered the understanding. Consequently 2008, Mattson, M.P. provided a comprehensive update and definition of hormesis^30^, while Calabrese, E.J. emphasized its significance^5^. In 2014, alongside the growth in publication volume, a highly influential work titled “ROS Function in Redox Signaling and Oxidative Stress,” authored by Schieber, M. et al., was also released. This article pioneeringly revealed the connection between hormesis and oxidative stress in redox biology, significantly broadening the scope and comprehension of hormesis research^49^.

In the region/country and institution analysis, it is evident that the United States and China are the top two countries with the highest publication output, with the United States having nearly four times the number of citations as China. The cooperation network map also shows strong linkages between the United States and other countries, solidifying its dominant position in the field of hormesis research. The University of Massachusetts, located in the United States Commonwealth of Massachusetts, emerges as the most influential institution, further supporting the US’s leading role in hormesis research. Notably, Supplementary Dataset 2 reveals that half of the top ten institutions in terms of publication output are based in China, with the Chinese Academy of Sciences ranking third in the total number of publications. This indicates that China is gradually gaining interest and prominence in this field, with the potential to become a significant research force in hormesis and inject new vitality into hormesis research^55,56^.

In the journal analysis, as shown in Supplementary Dataset 4, the top-ranked journal in terms of publication output is Dose–Response. Among the journals with high publication output and citations within the top 10 are PLOS ONE, Chemosphere, Science of The Total Environment, Environmental Pollution, and Human and Experimental Toxicology. Three of these journals belong to high-impact factor (JIF) journals in the JCR Q1 category. It is worth noting that although journals with higher publication output are mainly clustered in environmental science and toxicology, the top three journals with the highest total citations in the hormesis (PNAS, Science, and Nature) belong to top-tier multidisciplinary journals. This indicates that hormesis research holds significant value in academia, extending beyond its primary focus on pharmacology and toxicology and potentially having profound applications in fields such as biology and medicine.

In the author analysis, as shown in Supplementary Dataset 3 and Fig. 4, Calabrese E.J. consistently ranks first in total publication output, citations, citations, and link strength. He maintains a significant lead over the second-ranked author and exhibits considerable centrality in his connections with other authors in the field of hormesis. Furthermore, considering the results of the institution and journal analyses, Calabrese’s affiliation with the University of Massachusetts, where he ranks first in terms of total strength link, publication output, and citations, along with his role as the honorary editor of the influential journal Dose–Response, underscores his substantial impact. In the keywords clusters analysis, it is evident that Calabrese’s research since the late 19th century, tracing back to Hugo Schulz’s discovery of the hormesis concept^57,58^, has laid a solid foundation for the subsequent development of hormesis research. He has contributed to the consolidation and unification of terminologies used to describe hormesis in different fields^59^, provided a comprehensive and detailed definition of hormesis in terms of dose–response curve characteristics, underlying mechanisms, and effects^26^, and foresaw its generalizability and tremendous application prospects. While Calabrese E.J. mainly explores the value of hormesis in toxicology, biology, and medicine, Agathokleous focuses more on its relevance to environmental science and plant biology. Agathokleous ranks second in terms of publication output and total citations in the field, following closely behind Calabrese E.J. Their collaboration is also evident, with over 50 joint publications in the field over the past five years, where they have made forward-looking judgments regarding the characteristics of the linear non-threshold (LNT) dose–response model and the research potential of stimulants in the field of biology^60^. Additionally, it is worth noting from Supplementary Dataset 4 and Fig. 5 that although Ristow, Michael does not rank among the top ten authors in terms of publication output and total citations and occupies a relatively weak centrality position in the co-occurrence authors’ map, his total citations in the field of hormesis rank second, just behind Calabrese E.J. Four of his articles are featured in the top 15 most cited articles in the hormesis field (Supplementary Dataset 6). This indicates that Ristow, Michael might have discovered the phenomenon of hormesis from other research fields, such as metabolism. He has validated the existence of mitohormesis, which integrates the concept of hormesis with cellular metabolism, thereby establishing a deeper connection between hormesis and reactive oxygen species (ROS). In this process, he highlighted the crucial role of mitochondria, the primary cellular organelles responsible for metabolism. This further broadens the horizons of hormesis research and its applications, extending into the realm of physiology^61^. Ristow’s work has made substantial contributions to advancing hormesis research.

**Fig. 9:**
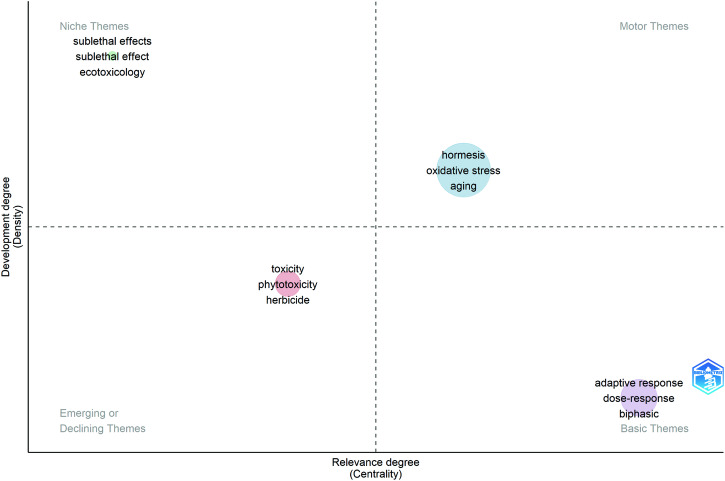
Analysis of research hotspots. The horizontal axis represents the relevance degree, while the vertical axis represents the development degree. The keywords in the upper-right quadrant have the highest relevance and the most vigorous development.

In the hotspots and frontiers analysis, Fig. 9 visually presents the occurrence and development of keywords in the field of hormesis. Combining the rankings of high-frequency keywords in Supplementary Dataset 5 and analyzing them with Fig. 6 can identify the field’s hotspots’ development trajectory and future trends. In the early stages of hormesis research, its primary value lay in studying and applying this toxic effect within the scope of plant biology. However, as the concept of hormesis and related ideas, such as homeopathy, gained acceptance and further research was conducted in the field of hormesis, the biphasic dose–response characteristics of hormesis and its connections with other stress responses gradually emerged. These discoveries expanded the application value of hormesis into physiology and medicine. Based on the characteristics of hormesis, moderate stimulation can induce various stress responses and enhance organism resistance, thereby exerting positive effects on the body. The relationship between oxidative stress and hormesis and the impact of hormesis on aging is currently a prevalent and emerging research direction. On the other hand, excessive stimulation can lead to adverse reactions in organisms, so the application of hormesis in the field of toxicology remains a topic worthy of close attention.

Based on the above analysis of current research on hormesis, we believe that exploring the mechanisms and applications of hormesis in toxicology, environmental science, and medicine will be promising research trends in the future. The most primary and urgent aspect is to provide authoritative definitions and interpretations of dose–response models for hormesis from a toxicological perspective. The establishment of dose–response models in hormesis serves as the foundation for various studies, which has been the focus of attention and discussion since the last century. These models describe the response processes and adaptation outcomes of cells and organisms to internal and external stressors or stimuli, holding significant importance in understanding and utilizing various sources of stress^62^.

The scientific community’s understanding of the dose–response model in hormesis could be more precise. It began with Muller’s establishment of the linear non-threshold (LNT) model for low-dose risk estimation^63^, and later Hugo Schulz proposed the biphasic dose–response model^64^. To this day, research on the dose–response model in hormesis continues to deepen. We now recognize that hormesis is essentially an “adaptive” regulatory response characterized by a biphasic dose–response curve^59^, and understanding the characteristics of this curve holds critical practical implications for applications in toxicology, pharmacology, and clinical medicine. One of the most significant quantitative features of this biphasic dose–response curve is the No-Observed-Adverse-Effect Level (NOAEL), which represents the highest dose at which the dose–response curve intersects with the control-response line, dividing the curve into two parts^65^. The left side of the NOAEL represents adaptive responses induced by low doses or sublethal effects. Hormesis can maintain homeostasis and enhance subsequent tolerance within this range^66^. In the long term, such responses improve organismal functions^67,68^. The right side of the NOAEL represents toxicity responses caused by excessive stress, where doses within this range lead to irreversible damage or even death. This observation is considered the most prominent and fundamental characteristic of hormesis induced by various stressors in the hormesis database established by Calabrese E.J. and Blain^69^, referred to as “a low dose stimulation and a high dose inhibition”^70^. Low-dose stimulation inspires the exploration of new measures for preventing and treating various diseases. At the same time, high-dose inhibition can be used for the removal of harmful substances both inside and outside organisms. For example, it can be utilized in developing novel herbicides through phytotoxicity, optimization of insecticides, or eradicating cancer cells.

**Fig. 10:**
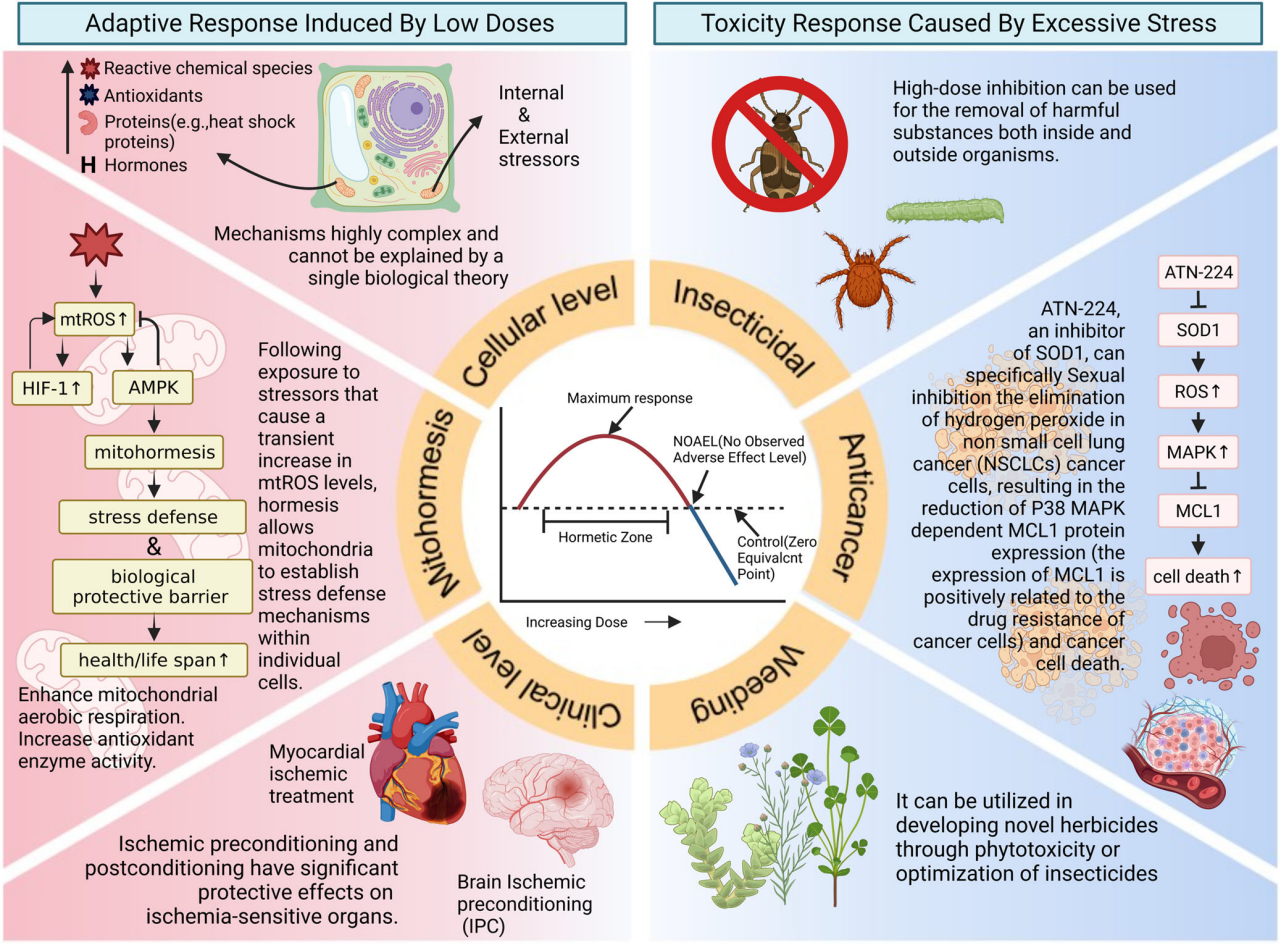
The connections and applications of hormesis in the fields of Environmental Science, Biology, and Medicine.

After completing a thorough analysis of the toxicological model of hormesis, the importance of hormesis to environmental science is also revealed and recognized. From Fig. 6 to Fig. 10, it is evident that the importance of hormesis in the 21st century extends consistently to the fields of environmental science and toxicology. It began with early research focused on whole effluent toxicity, ecological risk assessment^71,72^, and thermotolerance. Over time, this research expanded from a limited exploration of herbicides to the current understanding of ecotoxicity. Hormesis’s existence and value have been identified not only in plants, microorganisms, insects, and biological models but also in entire ecosystems^73^. Indeed, the ecosystem that sustains our existence has been undergoing constant changes throughout history, ranging from Earth’s environment with relatively low oxygen levels (only 5% of today’s levels) two hundred million years ago to the current era of global warming. This continuous change is undeniable and will likely persist, necessitating continuous evolution and adaptation of life on Earth to these changing environments^74^. Slight environmental changes can induce organisms to develop adaptive regulatory mechanisms to achieve a new equilibrium state. This concept is essential in understanding the significance of hormesis for the environment.

Environmental hormesis encompasses a range of stressors and biological endpoints^69^. Common physical stressors include habitat changes, seed priming^75^, high temperatures^76^, and drought. It is now recognized that the essence of the “optimal developmental temperature hypothesis” proposed by Zamudio et al. and Huey, R.B. in the last century^77^ is, in fact, hormesis. This hypothesis suggests that organisms exposed to mild heat stress perform better under subsequent high heat conditions than those exposed to excessive heat stress or no prior exposure to the stimulus^78^. Artificial chemical stressors like herbicides^79^ and paraquat^80^ were among the early substances discovered to induce hormesis. This phenomenon of adaptation to non-biological stressors was later found in various human/veterinary antibiotics. The large amounts of antibiotics used in intensive animal farming, when released into the environment through animal feces, were initially seen as ecotoxic environmental pollutants. However, it is now discovered that antibiotics-induced hormesis can activate plant defense mechanisms and enhance environmental adaptability at biochemical, growth, physiological, and production levels^17^, aligning with the “xenohormesis hypothesis” described by Howitz and Sinclair^81,82^. Chemical stressors in the natural environment also hold broad and widespread research value, including gaseous air pollutants and rare earth elements^83^. Ground-level ozone (O3), one of the most common air pollutants^84^, when absorbed by plants, disrupts their oxidative stress balance, causing severe economic losses and environmental pollution^85–87^. However, recent research has found effects of ozone beyond its phytotoxicity. The phenylpropanoids in hybrid poplar clones (*Populus our Americana* cv. ‘74/76’) can modulate the redox levels within organisms and enhance oxidative stress defense by responding to ozone-induced hormesis. Additionally, this effect is subject to modulation by soil nitrogen^88^.

A deeper understanding of hormesis mediated by chemical stressors could revolutionize our conventional understanding of environmental pollutants, offering a more comprehensive and objective approach to devising strategies for environmental improvement. Just as Agathokleous Evgenios proposed in the “How clean is clean?”^89^, We can anticipate that as our comprehension of hormesis expands to encompass more organisms and stress sources, it might be possible to transform the ecological toxicity of environmental pollutants into biological regulators. Exploring how environmental pollutants at low doses induce hormesis in organisms and even ecosystems, this shift in perspective could be instrumental in our efforts to better enhance, adapt to, and harness the environment.

After macroscopically analyzing the effects and application prospects of hormesis from the perspectives of environmental science and ecology, its micro-regulatory role in molecular biology, cell biology, and medicine has gradually been unveiled in recent years, becoming an increasingly hot research topic (Supplementary Dataset 5 and Fig. 9). The main focus of this research is on how to enhance physiological functions and develop therapeutic strategies during aging-related physiological processes or pathological conditions. As individuals age, their resistance and adaptation to various internal and external stressors decrease, leading to cumulative damage and an increased risk of diseases and tumors. The concept of hormesis unifies the mechanisms of numerous molecules and signaling pathways involved in lifespan regulation. Utilizing hormesis through active induction may improve aging processes^90^, making it a potential new target in aging research when combined with findings from other fields.

The connection between restricted diets and their beneficial effects on aging and hormesis was discovered in the last century^91–93^. Supplementary Dataset 5 and Fig. 9 demonstrate that the most prominent mechanism currently studied is oxidative stress related to reactive oxygen species (ROS). The understanding of ROS has expanded from the *Free Radical Theory of Aging* (FRTA)^94^, which describes cellular dysfunction, accumulation of harmful stimuli, and ultimately cell and organism death^95^, to encompass important signaling molecules regulating redox reactions^49,96,97^. This expansion has led to activating various adaptive responses in organisms to improve stress defense mechanisms^25^.

Therefore, more profound research has focused on mitochondria, the primary site of intracellular ROS generation. The term “mitohormesis” refers to the hormetic effects of ROS (referred to as mtROS) within mitochondria. Following exposure to stressors that cause a transient increase in mtROS levels, hormesis allows mitochondria to establish stress defense mechanisms within individual cells, such as enhanced mitochondrial aerobic respiration and increased antioxidant enzyme activity. This process, resembling clinical preconditioning, subsequently reduces overall cellular ROS levels, long-term maintenance of cellular homeostasis, and improved stress defense states^98^. When Caenorhabditis elegans is exposed to stress conditions such as hypoxia or mitochondrial respiratory inhibition, there is a transient increase in mitochondrial reactive oxygen species (mtROS) levels. This change induces an elevation in HIF-1 (hypoxia inducible factor-1) activity, which, through positive feedback regulation, mediates an increase in ROS production. Interestingly, the further increase in mtROS levels caused by this seemingly imbalanced process activates AMPK, which exerts a negative feedback effect, ultimately downregulating ROS levels until reaching homeostasis. Therefore, the orderly cascade reaction of AMPK/HIF-1 plays a vital role in regulating ROS levels. The upstream HIF-1 exerts an amplification effect. The ROS fluctuation caused by this process has been proven to be the reason for the extension of lifespan and enhanced immunity in *C. elegans*^99^.

Moreover, besides maintaining cellular homeostasis through the AMPK signaling pathway, recent studies by Calabrese V. et al. have highlighted the crucial regulatory roles of the NRF2 and nuclear factor-kappa B (NF-κB) pathways in the oxidative balance homeostasis of mitohormesis^100^. In response to elevated ROS levels, endogenous defense mechanisms in cells are activated, leading to the occurrence of cell adaptive stress responses mediated by the Keap1/NRF2/ARE pathway. This activation results in the transcription and expression of cytoprotective genes and essential antioxidant genes (vitagenes)^51,101–107^. The synthesis of cytoprotective proteins such as antioxidant enzymes, neurotrophic factors, heat shock protein 70 (Hsp70), heme oxygenase-1 (HO-1), among others, increases^107^. Additionally, the sirtuin protein system and glutathione redox system are activated, enhancing the organism’s resistance and adaptability to stress^108,109^. Moreover, the hormesis-mediated cellular defense mechanisms exhibit more prolonged protective effects compared to conventional antioxidants. This is particularly relevant for oxidative stress-sensitive neuronal cells, where their crucial role in neuroprotection may offer new research perspectives and therapeutic strategies for addressing age-related pathologies such as neuroinflammation, cognitive disorders, and age-related neurodegenerative diseases^110–116^. In addition, mitohormesis can also delay aging by promoting stem cell differentiation. It is known that stem cells undergo increased mitochondrial biogenesis and metabolism during differentiation, not only for energy demands but also for the role of ROS as signaling molecules in mediating cell differentiation. The differentiation of adipocytes, for example, requires activation of the PI3K signaling pathway and its downstream effector mTORC1, which mediates an increase in mtROS synthesis, thereby inducing the transcriptional mechanism of PPARγ required for initiating adipocyte differentiation^117^.

In addition to enhancing resistance and stress tolerance and promoting stem cell renewal to delay aging, hormesis also has significant research value in regulating cancer cells. Based on the biphasic dose–response characteristics of hormesis, the mechanisms by which ROS regulate tumors can be categorized into two specific analyses: reducing ROS levels to decrease cell growth-related signaling and inhibit cancer cell proliferation and increasing ROS levels to induce cancer cell death selectively. The former include tumor suppressor genes such as the forkhead box O (FOXO) family of transcription factors, which can inhibit tumorigenesis by inducing the expression of antioxidants^118^. The latter includes compounds like piperlongumine, which selectively activates oxidative stress responses in cancer cells by interacting with proteins that regulate oxidative stress, such as CBR1, GSTP1, and their complexes, thereby inducing cancer cell death under high ROS levels^119^. This selectivity makes piperlongumine more valuable and potentially applicable in research than traditional anticancer drugs such as paclitaxel and sulphoximine. Similar anticancer mechanisms include the SOD1 inhibitor ATN-224, which specifically inhibits the clearance of hydrogen peroxide within non-small-cell lung cancers (NSCLCs) cells, leading to a P38 MAPK-dependent decrease in MCL1 protein expression (MCL1 expression is positively correlated with cancer cell drug resistance) and cancer cell death^120^. ROS and other substances that regulate cell growth or act as signaling molecules can also serve as therapeutic targets for cancer treatment through hormesis mechanisms. For example, Weis et al. found that statin drugs commonly used in clinical practice for cardiovascular diseases, such as CEV/ATR (atorvastatin), can enhance inflammation-induced angiogenesis at low doses and inhibit tumor angiogenesis by promoting endothelial cell apoptosis at high doses, leading to a significant reduction in lung tumor development^121^. In addition to statins, other substances such as metformin^122^, ionizing radiation, resveratrol^123^, and other stress have been found to have similar anticancer effects within specific dose ranges. Apart from using the quantitative characteristics of hormesis curves to indirectly or directly inhibit cancer growth and proliferation as an anticancer approach, inducing distinct hormetic responses in normal cells and cancer cells can also enhance the effectiveness of cancer treatment. Combining short-term starvation or fasting with chemotherapy is an effective strategy to enhance the anticancer effects while minimizing damage to normal cells caused by chemotherapy^124^. Preoperative fasting can stimulate hormetic responses in normal cells, thereby enhancing the body’s resilience. However, due to genomic mutations in cancer cells, such as yeast cancer genes Ras and Sch9 (a functional orthologue of mammalian S6K)^125,126^, the ability to induce hormetic responses to increase adaptability is significantly reduced in cancer cells. This phenomenon is referred to as “differential stress resistance (DSR)”^127,128^. The protective effect of this anticancer adjunct approach on normal cells has been discovered in various tumors such as lymphomas^129^, breast cancer^130^, melanoma^131^, glioma, and neuroblastomas^124,128^.

Hormesis has far more applications in clinical practice than mentioned earlier. As pointed out by Calabrese E.J., due to the broad scope and extended period of research in this field, various proprietary terms are used to describe hormesis or related phenomena, and these terms have differed across different periods and disciplines. Currently, at least 30 terms exist, including inverted U-shaped, adaptive response, biphasic, low-dose stimulation, priming, and preconditioning^5^. These diverse proprietary terms can help us further understand the applications of hormesis in various medical research areas. Preconditioning and postconditioning are commonly used protective measures in clinical medicine, and both preconditioning and postconditioning are manifestations of hormesis. Therefore, hormesis can be induced in clinical practice through pre- and post-treatment measures to establish a biological protective barrier^132^. This approach enhances the body’s resistance and tolerance, preventing and alleviating damage caused by various diseases^133^. For example, ischemic preconditioning and postconditioning have significant protective effects on ischemia-sensitive organs. Preconditioning can reduce the likelihood of ischemic heart disease or decrease damage after myocardial ischemia reperfusion^134^; ischemic preconditioning (IPC) or transient ischemic accidents (TIAs) induce tolerance to cerebral ischemia and can reduce the severity of subsequent stroke^135,136^. Postconditioning, a relatively new concept, was initially identified as a low-level hypoxic stress response. It refers to the administration of a significantly reduced expected damage by applying a low-dose homologous or heterologous stimulus within a specific time window after a high-dose stimulus. Compared to preconditioning, although the research and clinical application of the former is more mature, its primary application fields are currently in public health or disease prevention. On the other hand, postconditioning can play a role in reducing the level of bodily damage, inhibiting disease occurrence and progression, and improving prognosis after various types of injuries or diseases^137^. For instance, the protective effect of remote ischemic postconditioning, similar to IPC (ischemic postconditioning), has been validated in a rat model of middle cerebral artery occlusion (MCAO) against brain ischemia^138^.

Recently, hormesis has also been introduced into the fields of infectious diseases and inflammation to explore new treatment approaches. Traditionally, sepsis was considered a condition of multiple organ dysfunction caused by an imbalanced host response to infection^139^. However, in 2021, Michael Bauer et al. proposed an innovative hypothesis, expanding the description of sepsis as “an illness state characterized by allostatic overload and failing responses of the organism to infection and other types of environmental stress”. This viewpoint suggests that excessive inflammatory responses, such as cytokine storms, in sepsis or septicemia can lead to multiple organ metabolic failure and mortality in acute sepsis patients. At the same time, immune tolerance and immunosuppression result in long-term damage to the immune system and organ function^140^. Therefore, understanding inflammation from the perspective of hormesis and selectively modulating the imbalanced state of the body could become a novel therapeutic direction. When endogenous factors such as levels of inflammatory cytokines within the body or exogenous stressors like pathogens are at relatively low levels, the immune function of the body gets activated, enhancing its adaptability through immune training. As the stimulus concentration increases further, immune tolerance is generated, which means that the body’s immune response level gradually decreases. Excessive inflammatory storms or severe inflammatory responses beyond the body’s regulatory capacity can disrupt the balance of regulatory levels and lead to immune exhaustion. Ultimately, irreversible damage to bodily functions can occur, such as in fatal conditions like septic shock or systemic inflammatory response syndrome (SIRS). In general, to fully regulate the body’s adaptability and harness its immune function, maintaining inflammatory responses within the hormetic zone could be a potential therapeutic approach. To counterbalance excessive pro-inflammatory responses or immune tolerance, the use of immunosuppressive agents and antiproliferative agents like rapamycin (an mTOR inhibitor)^141^ for inhibition is considered. Concurrently, patients with suppressed immunity might require the regulation of pro-inflammatory factors such as IFN-γ^142^ or enhancement of immune response capacity through induced immune training.

In conclusion, our review comprehensively analyzes the distribution of countries, regions, journals, and authors, along with the hot topics and development trends in hormesis research over the past 23 years. Our study offers a comprehensive overview of the fundamental concepts and current state of hormesis research, providing insights into potential research trends and frontiers. These findings will enhance understanding of hormesis across diverse fields and guide future investigations.

## Methods

### Data collection

Bibliometric studies often rely on the Web of Science Core Collection (WOSCC), a comprehensive, standardized database^143^, as it provides bibliometrics software with comprehensive statistical data, making it the most commonly used database for such analyses. In WOSCC, the abbreviation “TS” refers to “Topic Sentence”. The search query employed in this study was formulated as follows: “TS= (“Hormesis” OR “Hormeses” OR “Hormetic Dose–Response” OR “Dose–Response, Hormetic” OR “Dose-Responses, Hormetic” OR “Hormetic Dose–Response” OR “Hormetic Dose-Responses”). The study searched within the WOSCC using specific criteria. The search period was restricted to January 1, 2000, 2023. Only articles classified as “Article” or “Review” were considered, and the search was restricted to English-language publications. This approach resulted in a total of 3177 articles. The search results were exported in plain text (txt) and CSV formats, adhering to the designated search criteria. It is essential to mention that the search was conducted in 2023 to mitigate any potential bias stemming from database updates.

### Data analysis and visualization

VOSviewer and Citespace are commonly used software tools for bibliometric analysis, aiding researchers in importing statistical information from the Web of Science Core Collection. VOSviewer is an effective tool for visualizing literature, while Citespace assists researchers in creating and visualizing bibliometric maps, providing a visual understanding of the significance of various statistical data^144–146^.

We utilized the advanced visualization version of CiteSpace 6.1.R2 to conduct various analyses. These included examining the distribution and collaboration among countries, overlaying dual maps of journals, exploring the distribution of institutions, examining the disciplinary areas of the literature, analyzing the timeline of keywords, investigating cooperative relationships among references, and identifying literature bursts. We used VOSviewer 1.6.18 for conducting various bibliometric analyses, including the visualization of country distribution, institutional distribution, author distribution, collaborative relationships, and keyword cooperation. VOSviewer allowed us to analyze these networks by utilizing similarity matrices and automatic clustering, and we labeled the visualizations based on their content. To visualize the distribution of countries, references, and keywords, we utilized Bibliometrix, a tool available in R-Studio’s R-Tool. Publication and citation trends over the years were presented using Microsoft Excel 365. It is important to note that all the data used in this study were obtained from publicly available databases, so there was no need for ethical approval.

### Supplementary information

Supplementary Dataset 1

Supplementary Dataset 2

Supplementary Dataset 3

Supplementary Dataset 4

Supplementary Dataset 5

Supplementary Dataset 6

Data Availablity

Data Availablity

Data Availablity

Data Availablity

Data Availablity

Data Availablity

Data Availablity

Data Availablity

Data Availablity

Data Availablity

Data Availablity

Data Availablity

Data Availablity

Data Availablity

Data Availablity

Code Availablity

Data Availablity

Data Availablity

Data Availablity

Data Availablity

## Data Availability

The original contributions presented in this study are included in the supplementary information. Further inquiries can be directed to the corresponding authors.

## Code availability

The data that support the findings of this study are included in the supplementary information. Further inquiries can be directed to the corresponding authors.
